# Conjecture Regarding Posttranslational Modifications to the *Arabidopsis* Type I Proton-Pumping Pyrophosphatase (AVP1)

**DOI:** 10.3389/fpls.2017.01572

**Published:** 2017-09-12

**Authors:** Gaston A. Pizzio, Kendal D. Hirschi, Roberto A. Gaxiola

**Affiliations:** ^1^Center for Research in Agricultural Genomics, Consejo Superior de Investigaciones Científicas Barcelona, Spain; ^2^USDA ARS Children’s Nutrition Research Center, Baylor College of Medicine, Houston TX, United States; ^3^School of Life Sciences, Arizona State University, Tempe AZ, United States

**Keywords:** H^+^-PPase, AVP1, phosphorylation, sumoylation, ubiquitination, structural modeling

## Abstract

Agbiotechnology uses genetic engineering to improve the output and value of crops. Altering the expression of the plant *Type I Proton-pumping Pyrophosphatase* (H^+^-PPase) has already proven to be a useful tool to enhance crop productivity. Despite the effective use of this gene in translational research, information regarding the intracellular localization and functional plasticity of the pump remain largely enigmatic. Using computer modeling several putative phosphorylation, ubiquitination and sumoylation target sites were identified that may regulate *Arabidopsis* H^+^-PPase (AVP1- *Arabidopsis Vacuolar Proton-pump 1)* subcellular trafficking and activity. These putative regulatory sites will direct future research that specifically addresses the partitioning and transport characteristics of this pump. We posit that fine-tuning H^+^-PPases activity and cellular distribution will facilitate rationale strategies for further genetic improvements in crop productivity.

## Introduction

Constitutive expression of plant type I *Proton-pumping Pyrophosphatase* (H^+^-PPase) in crops improves several valuable traits including salt and drought resistance, shoot and root biomass and nutrient and water use efficiencies (Yang et al., 2007, 2014; Li et al., 2008; Bao et al., 2009; Pasapula et al., 2011; Pei et al., 2012; Arif et al., 2013; Paez-Valencia et al., 2013; Schilling et al., 2014; Wang et al., 2014). Currently more than 15 different crops have been improved using H^+^-PPase technology and in some cases these engineered plants demonstrate improved yield even in field conditions (reviewed in Gaxiola et al., 2016a,b; Schilling et al., 2017). The H^+^-PPases influences plant growth in both normal and abiotic stress conditions; however, how this protein alters growth has remained puzzling (Gaxiola et al., 2016a).

Fifteen years ago, the effects of H^+^-PPases were thought to be solely due to alterations around the vacuole (Gaxiola et al., 2001). The ability to buffer changes in the concentrations of essential and toxic ions requires judicious transport across the tonoplast (reviewed in Schumacher, 2014). This is energized by two proton pumps, the vacuolar H^+^-ATPase (V-ATPase) and the H^+^-PPase. V-ATPases are highly conserved, multisubunit proton pumps that consist of two subcomplexes. Increasing levels of V-ATPase activity has proven to be difficult because this is a complex of many proteins. However, the *Arabidopsis Vacuolar Proton-pump 1* (AVP1) transporter encodes a single polypeptide capable of enhancing the pumping of protons into the lumen of the vacuole (Kim et al., 1994). The simplicity of the structure made it an excellent candidate for manipulating proton gradients and this technology has been used in engineering numerous transgenic crops. Some of the improved growth in these engineered lines may be due to altered tonoplast transport as the salt-tolerant phenotype of transgenic lines expressing AVP1 or a homologue correlates in most of the crops tested with an increase in Na^+^ uptake into vacuoles (reviewed in Gaxiola et al., 2016a).

In the last several years, evidence has emerged that the H^+^-PPases is not solely localized to the vacuole and this pump may function as both a pyrophosphatase and as PP_i_-synthase (Pizzio et al., 2015; Gaxiola et al., 2016b; Khadilkar et al., 2016; Regmi et al., 2016; Schilling et al., 2017). In mesophyll cells the H^+^-PPase localizes at the tonoplast and with its PPi hydrolytic activity may serve two functions, vacuolar energization (Fuglsang et al., 2011 and references therein), and cytosolic PP_i_ scavenging (Ferjani et al., 2011). However, at the tonoplast it is possible that the H^+^-PPase can function as a PPi synthase depending of the vacuole pH. Evidence obtained from tonoplast fractions of maize coleoptiles and oranges suggests that a strong trans-tonoplast proton gradient affords this reverse PPi-synthase function (Rocha Facanha and de Meis, 1998; Marsh et al., 2000). The plasma membrane (PM) localization of H^+^-PPases is prominent in the sieve element-companion cell complexes (SE-CCs) in *Ricinus communis* and Arabidopsis (Paez-Valencia et al., 2011). In oxygen-deprived SE-CCs the PM localized type I H^+^-PPases may function as a PP_i_ synthase due to the prevailing trans-membrane proton-gradient (Paez-Valencia et al., 2011; Gaxiola et al., 2012; Tschiersch et al., 2012; Pizzio et al., 2015). Higher levels of PP_i_ favor Sucrose Synthase (SUS)-mediated Suc hydrolysis and respiration for the generation of ATP and the proton motive force (pmf) required for phloem Suc loading and long-distance transport (Paez-Valencia et al., 2011; Gaxiola et al., 2012, 2016b; Pizzio et al., 2015). This leads to speculation that the majority of phenotypes in H^+^-PPase-expressing transgenic crops may be due to increased PP_i_-synthase activity in SE-CCs to augment sucrose phloem loading and long-distance transport.

There are multiple scenarios that could explain the plasticity of the H^+^-PPases in terms of localization and activity. For example, a posttranslational modification could act as both a sorting signal and-or an activity switch. Alternatively, a protein chaperone could guide H^+^-PPase cell sorting and-or regulate its activity. Furthermore, a steep H^+^ gradient across the membrane may trigger the change of PPase to PPi-synthase activity (Marsh et al., 2000; Pizzio et al., 2015). Here we use computer modeling as a foundation to provide clues to identify regulatory elements within this protein that could impact trafficking and enzymatic functions. These *in silico* results will guide future experimental characterization of posttranslational modifications of the H^+^-PPase.

## Results and Discussion

### *In Silico* Prediction of Phosphorylation, Sumoylation and Ubiquitination Target Sites on AVP1

AVP1 appears to be localized at different membranes and may have multiple functions (Paez-Valencia et al., 2011; Gaxiola et al., 2012; Pizzio et al., 2015; Khadilkar et al., 2016). Using the AVP1 interactome provides clues to address how localization and activity are regulated. Using the on-line BIOGRID tool^1^ (Stark et al., 2006) several AVP1 interactors (**Table 1**) were identified including a putative protein kinase (AT1G07860; Jones et al., 2014), poly-ubiquitin 3 (UBQ3: AT5G3240; Manzano et al., 2008; Kim et al., 2013) and ubiquitin-conjugating enzyme E2 34 (UBC34: AT1G17280; Jones et al., 2014). Additionally, the SUMO-conjugating enzyme (SCE1: AT3G57870; Elrouby and Coupland, 2010) was found to interact with AVP1. These data imply that the H^+^-PPase could be regulated by phosphorylation, ubiquitination and/or sumoylation.

**Table 1:** AVP1 interactome.

Interactor	AGI	Description	Experimental evidence	Reference
UBQ3	AT5G03240	Polyubiquitin 3	Affinity Capture-MS	Manzano et al., 2008; Kim et al., 2013
CSP3	AT2G17870	Cold shock domain protein 3	Two-hybrid	Kim et al., 2013
SCE1	AT3G57870	SUMO-conjugating enzyme SCE1	Two-hybrid	Elrouby and Coupland, 2010
NHL3	AT5G06320	NDR1/HIN1-Like protein 3		
UBC34	AT1G17280	Ubiquitin-conjugating enzyme E2 34		
HHP2	AT4G30850	Heptahelical transmembrane protein2		
n.n.	AT2G39805	Integral membrane Yip1 family protein		
n.n.	AT1G14020	*O*-fucosyltransferase family protein	Protein-fragment complementation assay (PCA)	Jones et al., 2014
n.n.	AT1G07860	Putative protein kinase		
n.n.	AT1G47640	Hypothetical protein		
n.n.	AT3G66654	Cyclophilin-like peptidyl-prolyl *cis-trans* isomerase family protein		
n.n.	AT1G34640	Peptidase		

*Data extracted from BIOGRID. n.n., no-name*.

**FIGURE 1:**
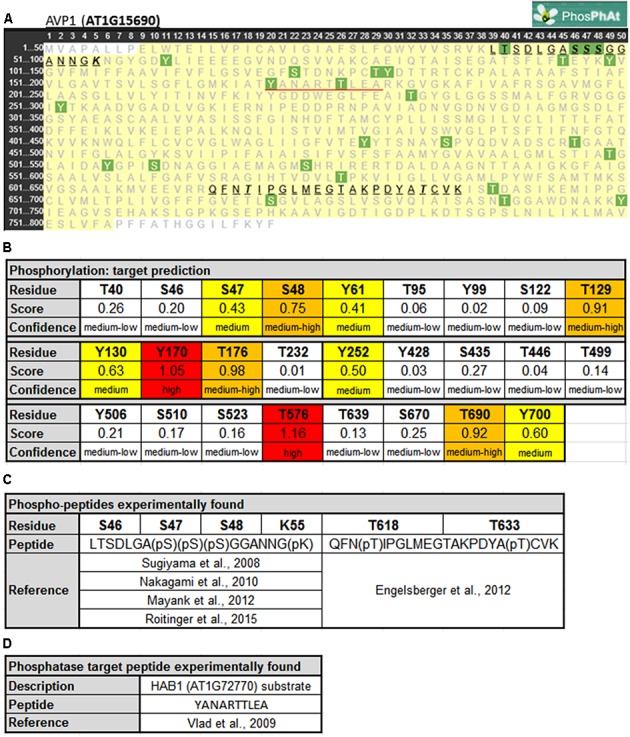
Predicted phosphorylation sites within AVP1. **(A)** Output given by PhosPhAt 4.0 (Durek et al., 2010; http://phosphat.uni-hohenheim.de/index.html). The residues in green are the phosphorylation targets. Phosphorylated peptides experimentally reported are highlighted and underlined in black. HAB1 target peptide is underlined in red. **(B)** Table with score and confidence of each phosphorylation target predicted. In red targets with high score values (<1), in orange medium-high score values (0.66 < score < 1), in yellow medium score values (0.33 < score < 0.66) and in white background medium-low score (0 < score < 0.33). **(C)** Peptides and phosphorylated residues reported in literature. **(D)** HAB1 substrate peptide reported before.

Protein phosphorylation is a fundamental mechanism through which protein function is regulated in response to extracellular stimuli (Champion et al., 2004). Using PHOSPHAT4.0^2^ (Durek et al., 2010), a specific protein phosphorylation target predictor for Arabidopsis, 26 different phosphorylation targets along AVP1 were identified (**Figures 1A,B**). Of particular note are residues Y170 and T576 (high score value), S48, T129, T176, and T690 (medium high score value), and S47, Y61, Y130, Y252, and Y700 (medium score value). Interestingly, two different AVP1-derived phospho-peptides were experimentally found in different approaches (**Figures 1A,C**). One of them (39-LTSDLGASSSGGANNGK-55) has a phosphorylation in S46, S47, S48 and/or K55 (Sugiyama et al., 2008; Nakagami et al., 2010; Mayank et al., 2012; Roitinger et al., 2015). A phosphorylation HOT-SPOT is defined as one containing 4 phosphorylatable residues within 10 consecutive amino-acids (PHOSPHAT 4.0; Durek et al., 2010). Furthermore, lysine (K55) may also act as a phosphate acceptor. It is well known that lysine can be targeted for one or more phosphoryl groups through a kinase phosphorylation or by a poly-phosphorylation mechanism (reviewed in Azevedo and Saiardi, 2016). Protein poly-phosphorylation at a lysine can be indirectly controlled by inositol pyrophosphate (Lonetti et al., 2011; Azevedo et al., 2015). In turn, inositol pyrophosphate is also involved in the regulation of cellular ATP levels (Szijgyarto et al., 2011; Wilson et al., 2013; Shears, 2015). We posit that AVP1 PPi-ase/PPi-synthase activity could be mediated by phosphorylation or poly-phosphorylation at K55.

The second AVP1-derived phospho-peptide (615-QFNTIPGLMEGTAKPDYATCVK-636) was experimentally described with a phosphate group at T618 and T633 (Engelsberger and Schulze, 2012). The modification at T618 was found when seedlings were grown under nitrogen starvation while the T633 modification was present during both adequate nutrition and nitrogen starvation conditions. A third AVP1-derived peptide (170-YANARTTLEA-179) is a substrate of the protein phosphatase HAB1 (AT1G7270; Vlad et al., 2009). Moreover, inside this peptide 170-YANARTTLEA-179 two residues (Y170 and T176) appear to be modified using the model generated by PHOSPHAT 4.0. Interestingly, HAB1 is a protein phosphatase involved in ABA signaling, a key hormone in abiotic stress response (Antoni et al., 2011). HAB1 may modify AVP1 under normal and abiotic stress conditions. These peptides (39-LTSDLGASSSGGANNGK-55, 615-QFNTIPGLMEGTAKPDYATCVK-636 and 170-YANARTTLEA-179) are unambiguously derived from AVP1 as they precisely match only this pump when BlastP was run against the *Arabidopsis* proteome (data not shown).

AVP1 interacts with the putative kinase AT1G07860 (Jones et al., 2014), and using NETPHOS 3.1 (Blom et al., 1999)^3^ others putative AVP1 kinases were identified. Several phosphorylation targets on AVP1 were predicted: S46, S47, S48, T176, Y252, T576, T633 and Y700 (Supplementary Figure 1). These targets were also predicted by PHOSPHAT (**Figure 1**). *Arabidopsis thaliana* encodes kinases related to Casein Kinase 1 (CKI), Cyclin-dependent Kinase 2 (cdc2), Protein Kinase C (PKC), Mitogen-activated Protein Kinase (MAPK) and the *trans*-membrane kinase Epidermal Growth Factor Receptor (EGFR) that could be mediating AVP1 phosphorylation (Supplementary Figure 1). These kinases are related with cell proliferation. In plant mitotic tissues PPi is produced in excess as a by-product of anabolism. It has been hypothesized that under these physiological conditions, the removal of PPi by H^+^-PPases favors both biosynthetic reactions and the energization of small vacuoles (Shiratake et al., 1997). Moreover, AVP1 working as a PPi-ase in early developmental stages (active mitotic tissues) is implicated in cytosolic PPi scavenging (Ferjani et al., 2011). Could phosphorylation on AVP1 (S46, S47, S48, T176, Y252, T576, T633 and Y700) be required to induce its PPase activity?

**FIGURE 2:**
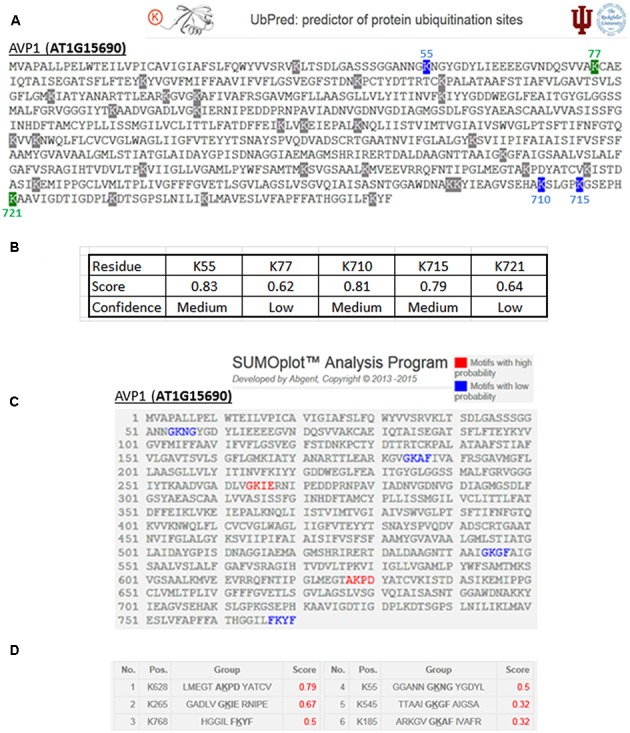
Predicted ubiquitination and sumoylation sites in AVP1. **(A)** Output given by UbPred (Radivojac et al., 2010; http://www.ubpred.org/). In green the predictions with low confidence and in blue medium confidence. Residues with gray have no confidence. **(B)** Score and confidence for each putative ubiquitination target. **(C)** Output given by SUMOplot (ABGENT; http://www.abgent.com/sumoplot/). In red the motif with high sumoylation probability and in blue low probability residues. **(D)** Table with the score assigned to each K sumoylation target prediction.

Ubiquitination regulates protein stability (Sadanandom et al., 2012; Sahara et al., 2014). Furthermore, ubiquitination has a role in protein localization, activation and protein–protein interactions (Varshavsky, 2006). For instance, ubiquitination regulates the protein dynamics of the plasma membrane-localized Brassinosteroids Receptor 1 (BRI1). A modified lysine residue impacts its internalization and tonoplast sorting (Martins et al., 2015). UbPred^4^ (Radivojac et al., 2010) predicts five ubiquitination targets on AVP1: K55, K77, K710, K715, and K721 (**Figures 2A,B**).

SUMOylation is able to modify proteins and is considered to be a major posttranslational regulator in plants (reviewed in Yates et al., 2016). For example, SUMOylation can regulate protein stability or interfere in protein–protein interactions (Wilkinson and Henley, 2010). The SUMOplot tool^5^ (ABGENT) was used to identify six sumoylation targets present in AVP1: K55, K185, K265, K545, K628 and K768 (**Figures 2C,D**). The sumoylation target predicted on AVP1 at residue K768 is within a key C-terminal loop. This loop may act as a H^+^ flux direction regulator throughout the transmembrane channel (Lin et al., 2012). The C-terminal loop of H^+^-PPases (a domain localized in the lumen of the vacuole) forms a hydrophobic gate in the proton transport pathway. In turn, this kind of gate could maintain unidirectional H^+^ translocation from the cytosol to the vacuolar lumen, avoiding H^+^ refluxing. Lin et al. (2012) propose this narrow pathway and its acid–base pairs as key regulators in the directionality of proton pumping flux of H^+^-PPases. Sumoylation at K768 could ‘lock’ this gate in an open conformation, and thus facilitate H^+^ refluxing and the PPi-synthase activity of the H^+^-PPase.

AVP1-K55 is not only included in the phosphorylation HOT-SPOT but also a possible phosphate acceptor and a putative target for ubiquitination and sumoylation. As a “mulitple-” target, AVP1-K55 could be an important residue that warrants further analysis.

### Structural Modeling of AVP1 and Topological Analysis of the Putative Posttranslational Modifications

**FIGURE 3:**
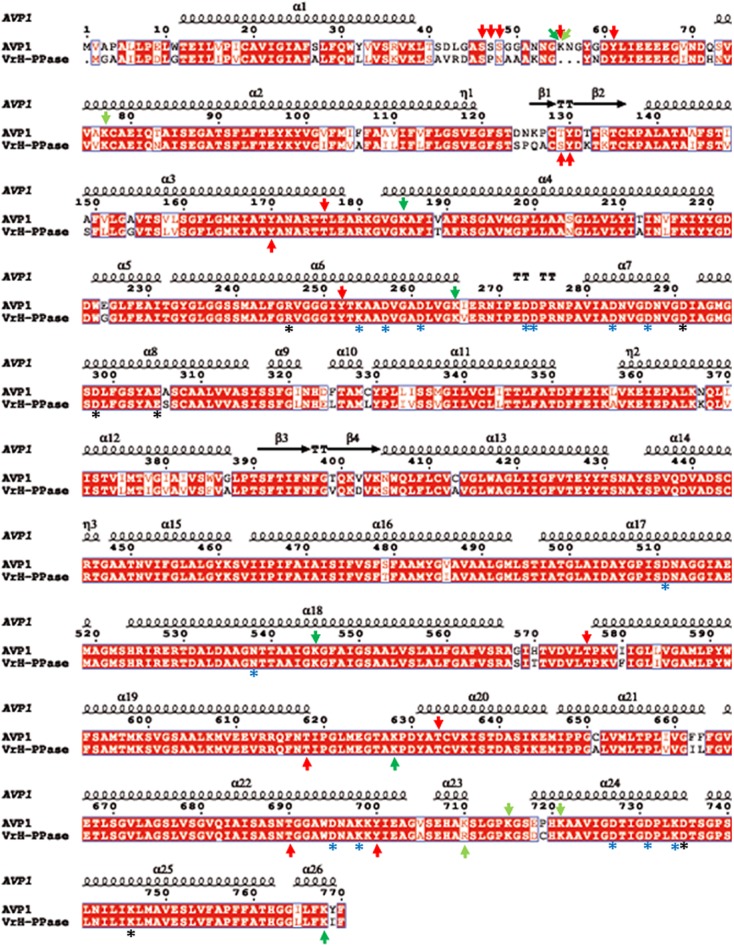
Alignment of AVP1 and VrH^+^-PPase. ESPript was used to align the two pumps (Robert and Gouet, 2014; http://espript.ibcp.fr/ESPript/ESPript/). Red arrows: phosphorylation targets; light green arrows: ubiquitination targets; dark green arrows: sumoylation targets. Black asterisk: key residues in the proton transport pathway. Blue asterisk: residues involved in PPi interaction.

**FIGURE 4:**
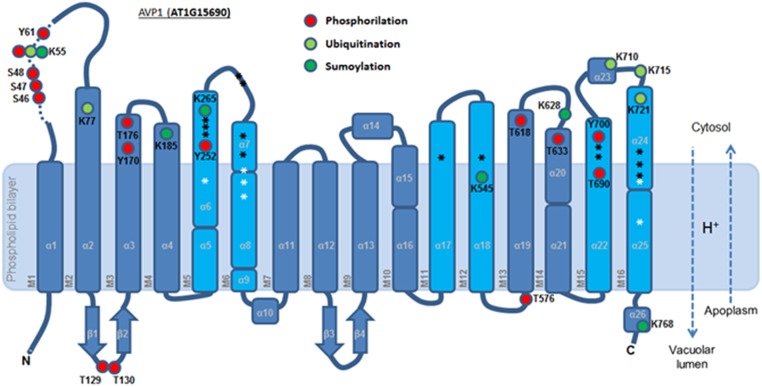
Predicted membrane topology of AVP1. The six inner (cyan) and ten outer (blue) transmembrane helices (M1-16). Red circle: phosphorylation targets. Light green circle: ubiquitination targets. Dark green circle: sumoylation targets. White asterisk: key residues in the proton transport pathway. Black asterisk: residues involved in PPi interaction. Dashed arrows: H^+^ flux direction.

**FIGURE 5:**
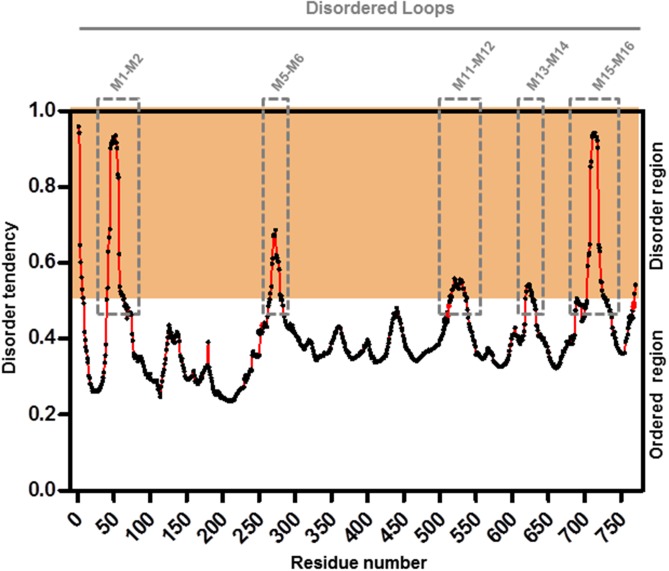
Meta-disorder prediction of AVP1. Local prediction of Intrinsically Unstructured Protein Regions (protein disorder) from amino acid sequence of AVP1 (GeneSilico MetaDisorder tool; Kozlowski and Bujnicki, 2012). All residues whose disorder probability is over 0.5 are considered as disordered. Method: MetaDisorderMD2 -CASP9 recommended by this tool as the most accurate disorder predictor method. Server: http://iimcb.genesilico.pl/metadisorder/metadisorder.html.

To further refine the relevance of putative posttranslational modifications in type I H^+^-PPases, protein modeling was performed. Given the lack of structural data on AVP1, we used the crystal structure of the homologous *Vigna radiata* H^+^-PPase (VrH^+^-PPase; Lin et al., 2012). To delineate the secondary structure of AVP1, alignment was performed between VrH^+^-PPase (primary and secondary structure) and the primary structure of AVP1 using EsPript^6^ (**Figure 3**). Given the high degree of amino acid sequence identity between H^+^-PPases (86–91% identity in land plants; Lin et al., 2012) this alignment (VrH1-PPase vs. AVP1) displayed high quality with protein identity at 88% and protein similarity at 94%. The putative posttranslational modification targets are present along the entire AVP1 sequence. Moreover, some of these targets (Y252, K265, K545, T690, Y700) are close to key AVP1 residues involved in PPi binding or H^+^ interactions inside the hydrophilic trans-membrane channel (**Figure 3**). The secondary structure predicted for AVP1 suggests all the putative posttranslational modifications, with the exception of K545 and T690 target amino-acids present in the cytoplasmic or apoplasmic/vacuolar loops (**Figure 4**). This is relevant because posttranslational modifications within trans-membrane domains are likely of little relevance. The HOT-SPOT (including S46, S47, S48 and K55) hits the unresolved region in the crystal structure of VrH^+^-PPase (M1-M2 loop; see **Figure 4**). Probably this region is not resolved in VrH^+^-PPase because it is an intrinsically disordered protein region (IDPR) and recalcitrant to crystallization (DeForte and Uversky, 2016). This idea is supported by the local disorder prediction of AVP1 sequence (**Figure 5**; GeneSilico MetaDisorder tool^7^; Kozlowski and Bujnicki, 2012) that predicts the amino-acid residues 40–63 of the M1-M2 loop are disordered. Interestingly, we found other IDPR or potentially flexible loops in AVP1 that include posttranslational targets: M5-M6 loop (including K265 and close to Y252); M11-M12 loop (close to K545), M13-M14 loop (including T618, K628 and T633) and M15-M16 loop (including T690, Y700, K710, K715, and K721). IDPR are associated with the domains’ ability to change its conformation and concomitantly the protein’s function (DeForte and Uversky, 2016). The primary sequence of a proteins or protein region encodes the ability to fold into an ordered functional unit or to stay intrinsically disordered but functional. IDPRs exist as dynamic structural ensembles and are involved in protein activity regulation through allosteric effects or posttranslational modifications that result in the masking and unmasking of interaction sites. (Bhowmick et al., 2013). IDPs are also abundant in protein degradation pathways. There are a number of E3 ubiquitin-protein ligases which have long stretches of disorder that appear to mediate interactions with a variety of mostly disordered substrates (Bhowmick et al., 2013; Erales and Coffino, 2014).

**FIGURE 6:**
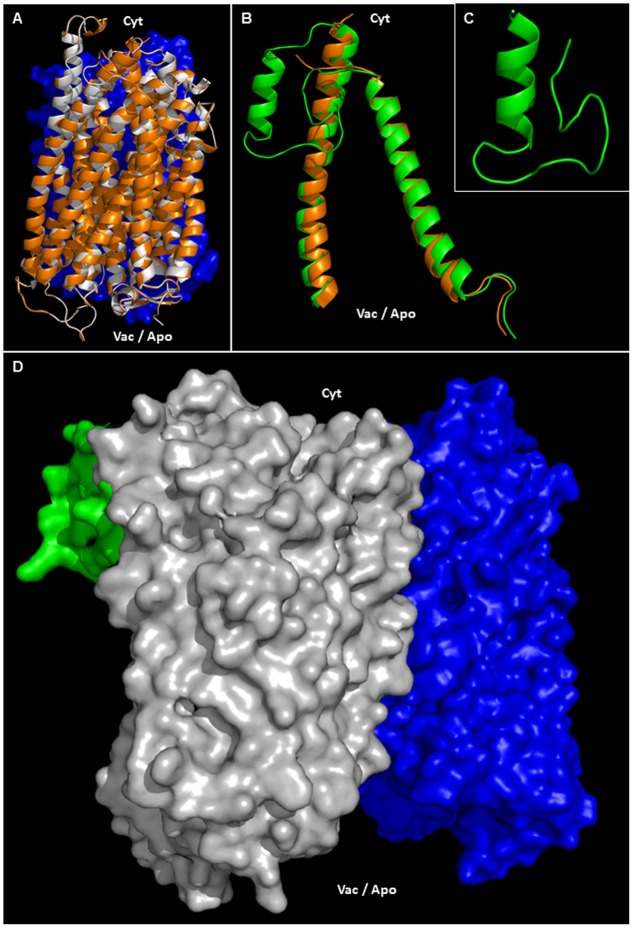
Structural modeling of AVP1. **(A)** Structural alignment of chain A VrH^+^-PPase (orange ribbons) and the putative structure of AVP1 (white ribbons). In blue: chain B VrH^+^-PPase surface. **(B)** Structural alignment of chain A VrH^+^-PPase_R1-100_ (orange ribbons) and the predicted structure of AVP1_R1-100_ (green ribbons). **(C)** Model of the AVP1 “flexible loop” (42-LGASSSGGANNGKNGYGDYLIEEEEGVND-71). **(D)** Protein surface of the H^+^-PPase homodimer: modeled AVP1 in white, AVP1 “flexible loop” in green and chain B VrH^+^-PPase in blue. Cyt: cytoplasmic side. Vac/Apo: vacuolar lumen and apoplasmic side.

Phosphorylation, ubiquitination or sumoylation are likely to occur at the protein surface in order to facilitate enzyme accessibility. Using PYMOD 2.0 (a plug-in for PYMOL software) with the crystal structure of A-VrH^+^-PPase as a template (Lin et al., 2012), AVP1 three dimensional models could be determined (**Figures 6A,D** and Supplementary Figure 2). AVP1 (white ribbons) and A-VrH^+^-PPase (orange ribbons) structural alignment displayed a high degree of similarity (**Figure 6A** and Supplementary Figure 2). The AVP1 structure was delineated with PYMOD/MODELLER by “Homology Based Modeling” using as a template VrH^+^-PPase (PDB: 4A01, resolved at 2.5 A°). AVP1 and the template VrH^+^-Pase are homologous proteins. They share more than 88% identity and 94% of similarity and for this reason the structural model is trustworthy (Baker and Sali, 2001; Zhang, 2009; Leman et al., 2015). Model assessment with DOPE local score (DOPE: Discrete Optimized Protein Energy; Shen and Sali, 2006; Webb and Sali, 2014) given by PYMOD/MODELLER showed high correlation between the AVP1 model (green line) and the VrH^+^-PPase crystal structure (blue line; Supplementary Figure 3A). The gap in VrH^+^-Pase DOPE score corresponded with the structural indel (protein internal deletion) defined as a “flexible loop” and is not resolved in the crystal structure. Ramachandran plot analysis that facilitates a visualization of energetically allowed regions for backbone dihedral angles ψ against φ of amino acid residues in protein structure (Ramachandran et al., 1963; Richardson, 1981) demonstrated the absence of any amino acid residue in outlying regions (Supplementary Figure 3B). Moreover, global quality *Z*-scores (QMEAN6 *Z*-score: -2.41, All atom: -1.73, Cbeta: -2.18, Solvation: 1.59, Torsion: -2.71, SS Agree: -1.56 and ACC Agree: -0.13) suggest the AVP1 structural model is reliable (Supplementary Figure 3C; SWISS-MODEL QMEAN tool; Studer et al., 2014). QMEAN “local” quality score shows almost all amino acid residues had a high score (near to 1). As expected, residues present in the “flexible loop” demonstrated a poor local quality score (Supplementary Figure 3D). To delineate the structure of this flexible loop (41-VRDASPNAAAKNGYNDYLIEEEEGIND-67 in VrH^+^-PPase and 42-LGASSSGGANNGKNGYGDYLIEEEEGVND-71 in AVP1) a partial AVP1 modeling (residues 1–100) was done using PHYRE2 (Protein Fold Recognition Server^8^; Kelley et al., 2015). Multi-template “Homology Based” and “AB initio” modeling where applied by PHYRE2. VrH^+^-PPase (PDB: 4A01) as the main template and used to model AVP1-residues 1–100 (70% modeled at > 90% confidence). AVP1 helix M1 and M2 (see **Figure 5**) appear to anchor the flexible loop’s extremities. In particular, the flexible loop N-terminal fragment (LGASSSGGANN) was modeled by AB initio and the C-terminal fragment (GKNGYGDYLIEEEEGVND) was delineated by homology base modeling: using a fragment of PDB-2N0Y as a partial secondary template (with 39% identity respect to AVP1). A Ramachandran plot of the flexible loop demonstrated only one amino acid residue in an outlying region (Supplementary Figure 4A). Moreover, global quality *Z*-scores (QMEAN6: -2.16, All atom: -1.63, Cbeta: -3.13, Solvation: -1.10, Torsion: -1.76, SS Agree: -0.90 and ACC: -0.11) again suggest that our model of the AVP1 flexible loop is dependable (Supplementary Figure 4B). Flexible loop modeling indicated a new alpha-helix (**Figures 6B,C**). The structural alignment of AVP1-residues 1–100 (green ribbons) and the A-VrH^+^-PPase chain (orange ribbons) displayed little variation (**Figure 6B**). A structural alignment of both protein fragments, AVP1 and the flexible loop, facilitates a model of the whole AVP1 surface (**Figure 6D**; as white surface AVP1 and as green surface the flexible loop).

**FIGURE 7:**
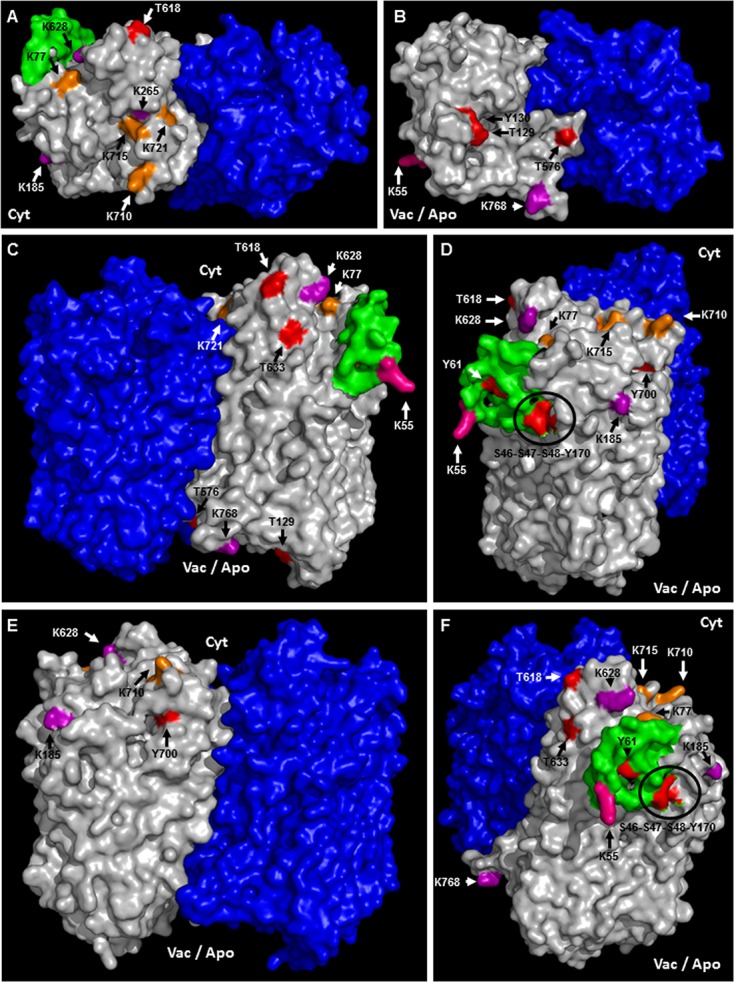
Predicted membrane topology and posttranslational modifications in AVP1. **(A–F)** Different views (protein surface) of the H^+^-PPase homodimer: modeled AVP1 in white, AVP1 “flexible loop” in green and chain B VrH^+^-PPase in blue. Phosphorylation targets in red. Ubiquitination targets in orange. Sumoylation targets in purple. Triple target in pink. Cyt: cytoplasmic side. Vac/Apo: vacuolar lumen and apoplasmic side.

A topological analysis of AVP1 structure shows that the phosphorylation targets S46, S47, S48, K55, Y61, T129, Y130, Y170, T576, T618, T633, and Y700, the ubiquitination targets K55, K77, K710, K715, and K721, and the sumoylation targets K55, K185, K265, K628, K768, are all on the protein surface (**Figures 7A–F** and Supplementary Figure 5). Thus, this topological analysis reinforces the potential relevance of these sites. Meanwhile, the phosphorylation sites T176, Y252 and T690, and the ubiquitination site K545 are buried inside the protein (Supplementary Figure 5), making these sites less likely to be important in protein regulation. Alternatively, the structure of this protein may be in dynamic flux with conformational changes being regulated by different modifications.

### Conclusions

AVP1 has been widely used in agbiotechnology to increase crop yield. Future basic science should be undertaken to guide AVP1 mediated engineering approaches. Our results suggest work can now be directed at understand the relevance of residues: S46, S47, S48, K55, Y61 because this is a phosphorylation HOT-SPOT; K55 could in turn also be ubiquitinated or sumoylated; Moreover, Y170 can be investigated as a target for the phosphatase HAB1. K265/T690/Y700 are proximal to putative active sites in the protein and may help regulate functional plasticity. Other work can examine if T618 is involved in regulation under nitrogen starvation. Lastly, K768 is of particular interest since it could regulate the directionality of H^+^ flux. This basic biology will shed light on AVP1 intracellular localization and activity allowing more rationale strategies to improve crop performance.

## *In Silico* Tools and Software

### BioGRID

Biological General Repository for Interaction Datasets (Stark et al., 2006). BioGRID is an interaction repository with data compiled through comprehensive curation efforts. Server at: http://thebiogrid.org/

### PHOSPHAT 4.0

Phosphorylation site database and predictor specific for*Arabidopsis* (Durek et al., 2010). Server at: http://phosphat.uni-hohenheim.de/index.html.

### BlastP

The Basic Local Alignment Search Tool for proteins (Johnson et al., 2008). Programs search protein databases using a protein query Server at: https://blast.ncbi.nlm.nih.gov/Blast.cgi?PAGE=Proteins.

### NetPhos 3.1

Predicts serine, threonine or tyrosine phosphorylation sites in eukaryotic proteins using ensembles of neural networks (Blom et al., 1999). Both generic and kinase specific predictions are performed. Predictions are made for the following 17 kinases: ATM, CKI, CKII, CaM-II, DNAPK, EGFR, GSK3, INSR, PKA, PKB, PKC, PKG, RSK, SRC, cdc2, cdk5 and p38MAPK. Server at: http://www.cbs.dtu.dk/services/NetPhos/.

### UbPred

Predictor of protein ubiquitination sites (Radivojac et al., 2010). Server at: http://www.ubpred.org/. UbPred is a random forest-based predictor of potential ubiquitination sites in proteins. It was trained on a combined set of 266 non-redundant experimentally verified ubiquitination sites.

### SUMOplot

Predicts and scores sumoylation sites in a protein (ABGENT). Server at: http://www.abgent.com/sumoplot/.

### EsPript 3.0

Easy Sequencing in PostScript (Robert and Gouet, 2014). Server at: http://espript.ibcp.fr/ESPript/ESPript/. EsPript is a program which renders sequence similarities and secondary structure information from aligned sequences for analysis and publication purpose.

### GeneSilico MetaDisorder

Local prediction of Intrinsically Unstructured Protein Regions (protein disorder) from amino acid sequences (Kozlowski and Bujnicki, 2012). Method: MetaDisorderMD2. Server at: http://iimcb.genesilico.pl/metadisorder/metadisorder.html.

### PyMol 1.6 Software

The PyMOL Molecular Graphics System, Version 1.6 Schrödinger, LLC^9^.

### PyMod 2.0 Software

PyMod 2.0 is a PyMOL plugin (Janson et al., 2016). PyMod was designed to act as simple and intuitive interface between PyMOL and several bioinformatics tools (i.e., PSI-BLAST, Clustal Omega, MUSCLE, CAMPO, PSIPRED, and MODELLER). DOPE score, or Discrete Optimized Protein Energy, is a statistical potential used to assess homology models in protein structure prediction. DOPE is based on an improved reference state that corresponds to non-interacting atoms in a homogeneous sphere with the radius dependent on a sample native structure; it thus accounts for the finite and spherical shape of the native structures. Alternatively, DOPE can also generate a residue-by-residue energy profile for the input model, making it possible for the user to spot the problematic region in the structure model. (Shen and Sali, 2006; Webb and Sali, 2014).

### Phyre2

Protein Fold Recognition Server (Kelley et al., 2015). Server at: http://www.sbg.bio.ic.ac.uk/phyre2/html/page.cgi?id=index. The Phyre2 is a web portal for protein modeling, prediction and analysis.

### RAMPAGE

Ramachandran plot analysis tool (Lovell et al., 2003). Tool for visualization of energetically allowed regions for backbone dihedral angles ψ against φ of amino acid residues in protein structure (Ramachandran et al., 1963; Richardson, 1981). Server at: http://mordred.bioc.cam.ac.uk/~rapper/rampage.php.

### SWISS-MODEL QMEANbrane

QMEAN is a composite scoring function based on different geometrical properties and provide a global absolute quality estimates on the basis of one single model. QMEANbrane is a QMEAN function specific for membrane proteins. The QMEAN *Z*-score provides an estimate of the ‘degree of nativeness’ of the structural features observed in the model. Higher QMEAN *Z*-scores indicate better model structure (Studer et al., 2014). Server at: https://swissmodel.expasy.org/qmean/.

## Author Contributions

Conception of the research, analyzing data, manuscript draft and final approval: GP, KH, and RG; bioinformatics: GP.

## Conflict of Interest Statement

The authors declare that the research was conducted in the absence of any commercial or financial relationships that could be construed as a potential conflict of interest.

## Footnotes

1 http://thebiogrid.org/

2 http://phosphat.uni-hohenheim.de/index.html

3 http://www.cbs.dtu.dk/services/NetPhos/

4 http://www.ubpred.org

5 http://www.abgent.com/sumoplot/

6 http://espript.ibcp.fr/ESPript/ESPript/

7 http://iimcb.genesilico.pl/metadisorder/metadisorder.html

8 http://www.sbg.bio.ic.ac.uk/phyre/

9 http://pymol.org/
