# Supplementary figures and images for: Conjecture Regarding Posttranslational Modifications to the Arabidopsis Type I Proton-Pumping Pyrophosphatase (AVP1)
Source: Front Plant Sci. 2017 Sep 12;8:1572. doi: 10.3389/fpls.2017.01572 (PMC5601048; doi:10.3389/fpls.2017.01572)

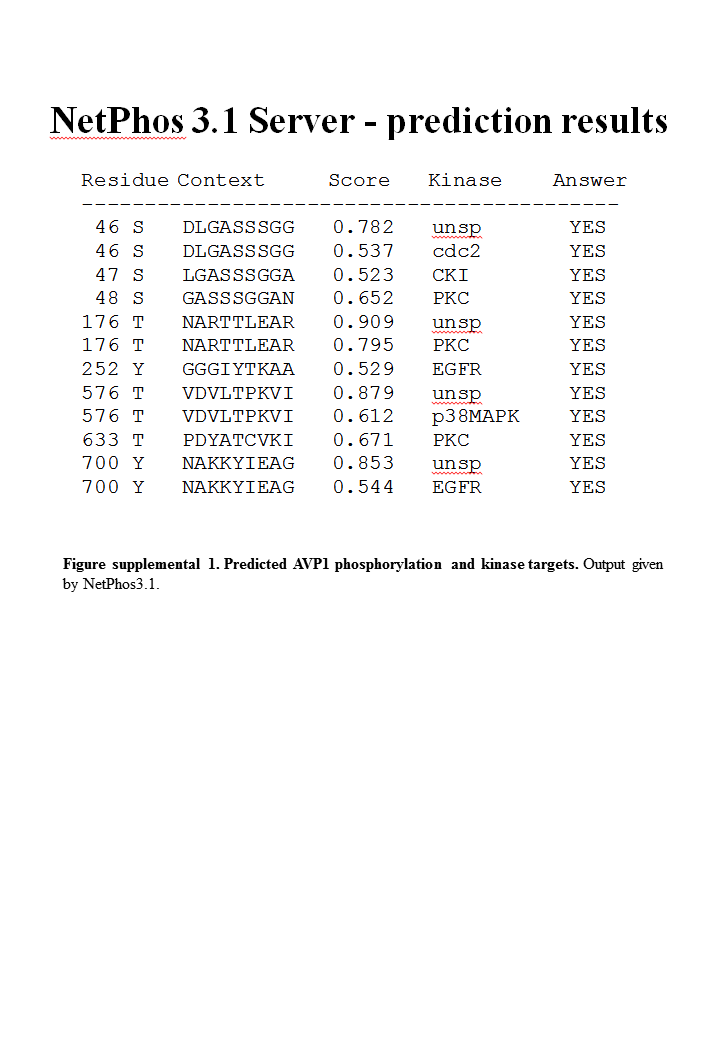

Supplement: Supplementary file 1 [file Image_1.TIF]

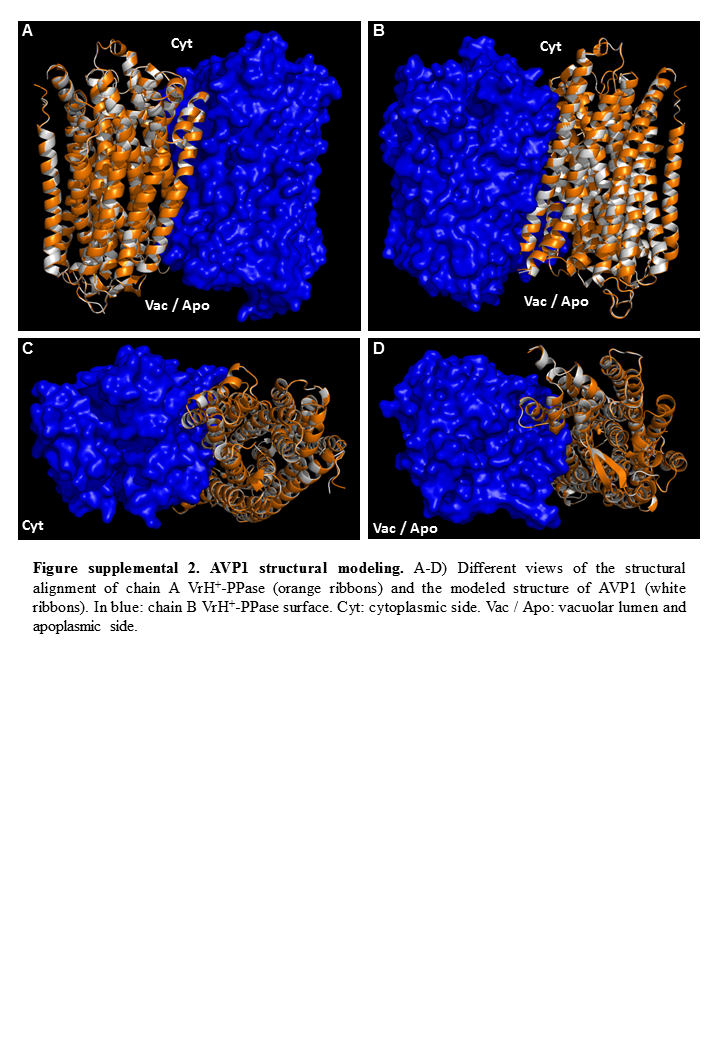

Supplement: Supplementary file 2 [file Image_2.tif]

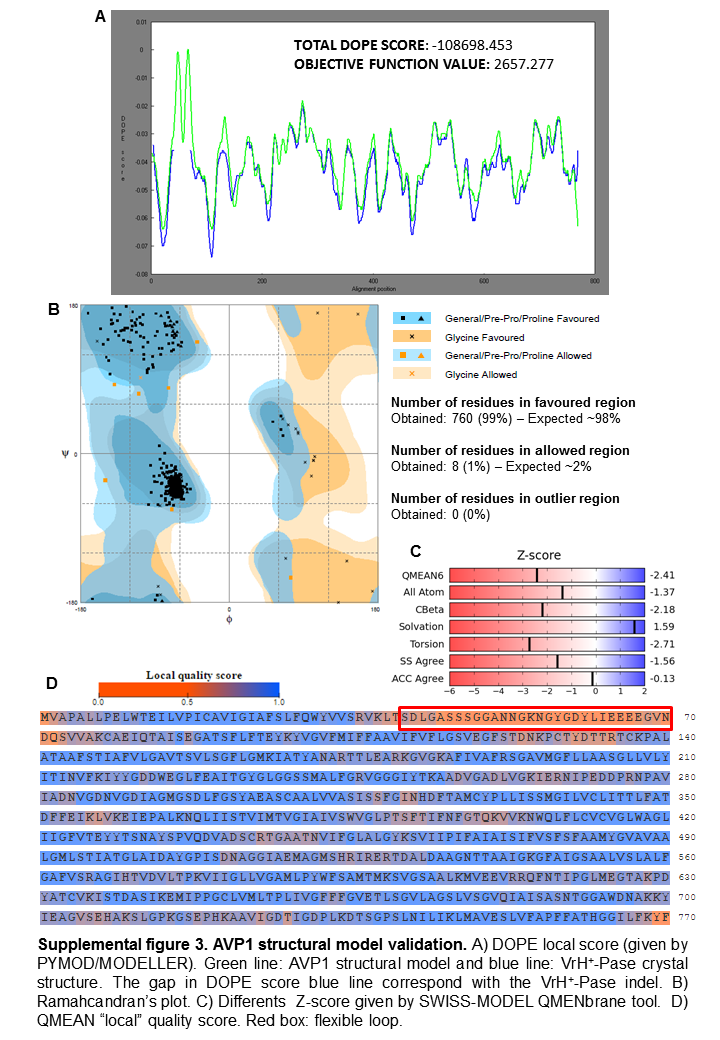

Supplement: Supplementary file 3 [file Image_3.tif]

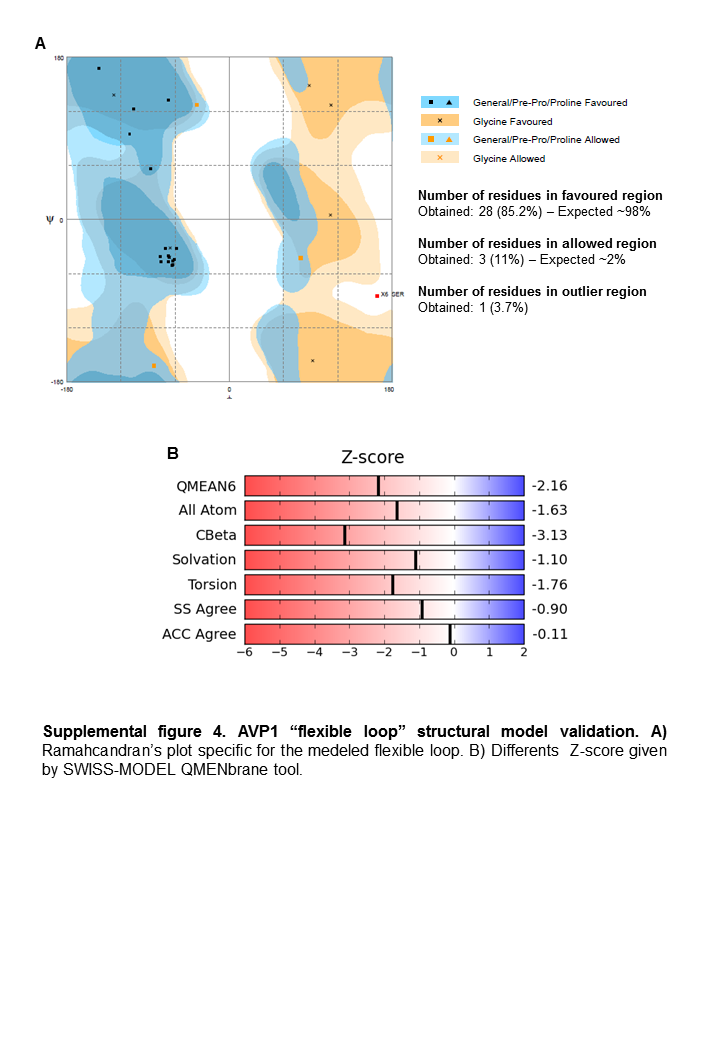

Supplement: Supplementary file 4 [file Image_4.tif]

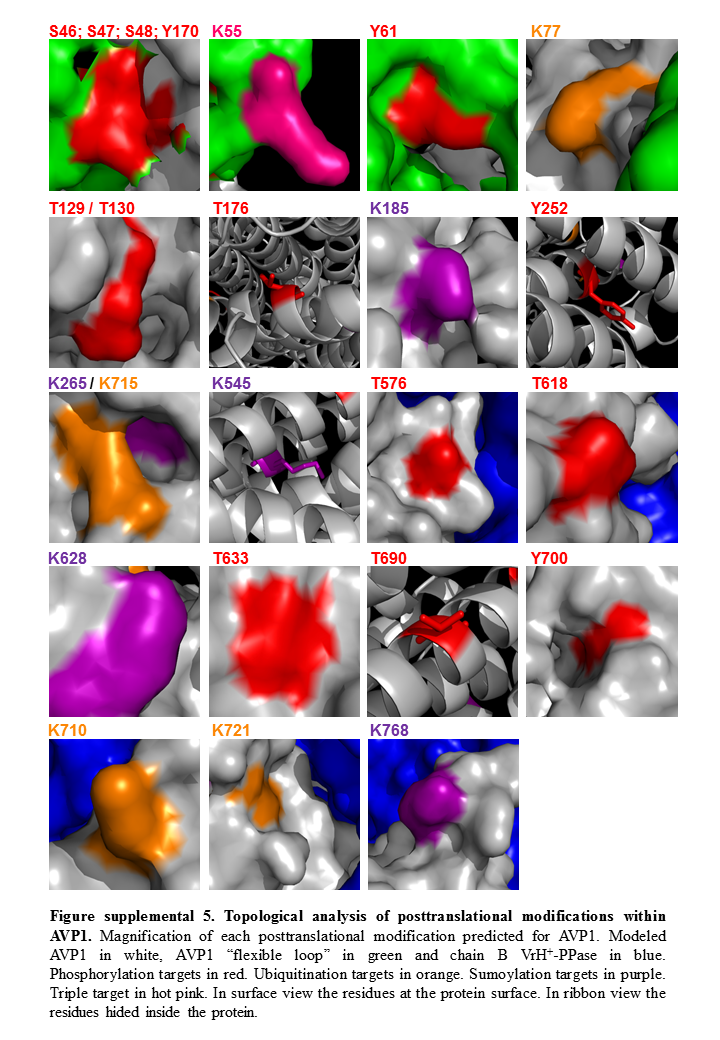

Supplement: Supplementary file 5 [file Image_5.tif]
